# Spatiotemporal Distribution of Malaria in the Kingdom of Saudi Arabia

**DOI:** 10.3390/tropicalmed9010016

**Published:** 2024-01-08

**Authors:** Ahmed Elagali, Mosa Shubayr, Elsiddig Noureldin, Kefyalew Addis Alene, Asmaa Elagali

**Affiliations:** 1 School of Biological Sciences, University of Western Australia, Perth, WA 6009, Australia; 2 Department of Zoology, Omdurman Islamic University, Al Khartoum 14415, Sudan; asmaalagali@gmail.com; 3 Department of Preventive Dental Sciences, Jazan University, Jazan 45142, Saudi Arabia; 4 Veterinary Laboratory of Tropical Diseases, Jazan 45142, Saudi Arabia; siddignoureldin@hotmail.com; 5 Faculty of Health Sciences, Curtin University, Perth, WA 6102, Australia

**Keywords:** malaria, epidemiology, spatiotemporal distribution, ecological determinants, Kingdom of Saudi Arabia

## Abstract

Background: Malaria is a significant public health concern in the Kingdom of Saudi Arabia (KSA). This study aimed to investigate the spatiotemporal distribution of malaria in the KSA between 2017 and 2021. Methods: A spatial analysis was conducted using data for malaria cases stratified by *Plasmodium* species reported by the Ministry of Health for the period 2017–2021. Covariate data such as environmental, socioeconomic, and demographic factors were assembled from different publicly available sources. Results: A total of 13,852 cases were reported from 20 regions in the KSA during the study period. The study indicated a decline in the overall number of reported cases from 2715 in 2017 to 2616 in 2021, primarily driven by a decrease in *Plasmodium falciparum* infections. However, the number of *Plasmodium vivax* cases increased in 2021. Southern regions of the KSA remained at higher risk due to imported cases from neighboring Yemen. Socioeconomic and demographic factors, such as access to healthcare and education, were found to affect malaria transmission. Environmental factors, such as temperature and rainfall, were also identified as determinants of malaria risk. Conclusions: This study showed significant spatial variation in malaria cases in the KSA that was related to underlying socioeconomic status and environmental factors. The findings of this study highlight the need for continued efforts to control and eliminate malaria in the KSA, particularly in regions with higher risk of malaria.

## 1. Introduction

Malaria is a mosquito-borne disease that can be a life-threatening illness and is a serious public health concern across the world, particularly in tropical and subtropical countries [1,2,3]. Malaria is endemic in over 90 countries, impacting over 40% of the global population. Five known parasite species can cause malaria infection, namely *Plasmodium falciparum*, *Plasmodium knowlesi*, *Plasmodium ovale*, *Plasmodium vivax*, and *Plasmodium malariae* [3]. Acute malaria infection is mostly caused by *P. falciparum*; hence, it is the primary parasite species responsible for malaria deaths [1]. The World Health Organization (WHO) reports an estimated 608,364 deaths in 2022 due to malaria infection, 1% less than in 2021 but still 6% more than in 2019 before the COVID-19 outbreak [1,2,3,4]. Malaria cases increased in 2021 but at a slower rate than in 2020. In 2021, there were 247 million reported cases, up from 245 million in 2020 and 232 million in 2019 [1,2,3]. Service interruptions due to the COVID-19 pandemic have hampered access to important malaria interventions across endemic countries, accounting for a major share of the increased malaria mortality and morbidity [5]. These disturbances resulted in an additional 63,000 malaria mortality and 13 million cases over the two peak COVID-19 pandemic years (2020–2021).

Malaria morbidity is most prevalent in African nations (92%), followed by South-East Asian countries (5%) and Eastern Mediterranean countries (2%). Nearly 80% of all malaria deaths occurred in 15 countries, the majority of which were in Africa. Since the time before Islam (610 CE), people have been aware of malaria across the Arabian Peninsula, notably in the Kingdom of Saudi Arabia (KSA). The Arabian American Oil Corporation (ARAMCO) started implementing a program to control malaria in the KSA in 1948 in the Eastern region, mostly to protect the local workforce from severe malaria infections. A few years later, in 1952, the Saudi government used this program as the basis for a countrywide malaria campaign and targeted malaria-prone areas all around the country [6]. The KSA joined the WHO’s global malaria eradication campaign in 1963, and ten years later, with exceptional success, the transmission of malaria infection had been stopped in the Eastern and Northern regions, eradicating malaria in the Palaearctic ecozone [7]. As a result, malaria transmission has stopped or has low frequency in the northern and eastern regions, particularly in urban areas [8]. However, malaria remains endemic in the Aseer and Jazan regions [9,10,11]. The national malaria control program has faced some obstacles in the past few years due to migration, climate change, documentation and communication methods, and a lack of trained workforce in these regions [11].

In the KSA, more than a thousand positive malaria cases have been reported each year since 2000, with the majority of cases caused by *P. falciparum*. The highest number of positive cases are normally reported in Jazan [9,11], with the fewest or no cases reported from Qurrayaat region [8]. For instance, in 2016 there were 5382 malaria cases recorded in the KSA; however, only 272 cases were locally acquired (indigenous), and all were *P. falciparum* infections. *P. falciparum* accounted for 72.9% of all malaria cases, *P. vivax* for 26.4%, and *P. malariae* for 0.7%. All of the locally acquired cases were from the south-west (Jazan and Asser), whereas the imported cases were mostly from Yemen, India, Pakistan and Sudan. Moreover, nearly 2 million pilgrims from approximately 65 countries worldwide visit Makkah during the annual Hajj season [12]. Malaria is endemic in some of these nations, particularly in Africa and Asia, including but not limited to India and Pakistan in Asia and Nigeria in Africa. This results in a higher occurrence of imported cases in the KSA. The annual incidence rate of malaria in the KSA varies significantly based on population migration and the timing of substantial rains, with transmission reaching its peak between October and March.

The epidemiology of malaria in the KSA varies from region to region and even from place to place within the same region, due to the vast area of the country and the variation in environmental conditions. These conditions play a crucial role in the distribution of malaria by affecting mosquito species, their life cycle, and the distribution abundance of mosquitoes. The Kingdom is home to seventeen different species of *Anopheles*, four of which, *An. arabiensis*, *An. sergentii*, *An. stephensi*, and *An. superpictus*, are recognized as capable malaria vectors. The less effective vectors in the east (*An. stephensi*), the center and west (*An. sergentii*), and the north (*An. superpictus*) are more susceptible to vector control in contrast to the robust and more effective vector *An. arabiensis* in the south-western region. The most common vector in the country is *An. sergentii*, and it has been connected to transmission of malaria infections in the south-west along with *An. arabiensis* [13].

In this study, we examined the epidemiology, distribution, trends, and ecological determinants of malaria risk in the KSA between 2017 and 2021 to offer valuable insights into the then-current state of malaria in the Kingdom. This included the reported number of cases, the species of *Plasmodium* responsible for the majority of cases, and the regions where the disease remained endemic. The study also explored how environmental factors influenced the distribution of malaria in the KSA and how those factors impacted the observed patterns during the study period.

## 2. Materials and Methods

### 2.1. Study Settings

The study was conducted in the KSA, which is situated at the farthest point of Southwestern Asia. It shares its borders with the Arabian Gulf, the United Arab Emirates, and Qatar to the east; the Red Sea to the west; and Kuwait, Iraq, and Jordan to the north. In the south, it is bordered by Yemen and Oman (https://www.stats.gov.sa/en/page/259, accessed on 1 Jannuary 2024). Riyadh serves as the capital city of the Kingdom of Saudi Arabia, which holds the distinction of being the largest country on the Arabian Peninsula. The KSA’s terrain encompasses deserts, mountain ranges, and grasslands, resulting in diverse climates across different regions. As of 2021, the estimated population of the KSA is around 32.1 million people, with 58.4% being Saudi nationals and 41.6% foreigners, with approximately three-fifths of the population in the KSA residing in major cities in the country [14,15].

The kingdom may be split into two natural zones: the rain-fed highlands of the western and southwestern regions (Sarawat Mountains) and the vast desert and extra-arid plains of the interior (Najd). Despite its desert climate, the climatic conditions in the KSA vary by location owing to the country’s diversified terrain. The desert center of the country has dry hot summers and cold winters, while the coastal areas have high temperatures and humidity, and the southwestern half of the country has a moderate climate. Perennial mountain streams, on the other hand, provide water for various lush valleys and oases in the Kingdom’s southern regions, where human groups and vector populations cohabit and the bulk of malaria cases are reported.

### 2.2. Data Sources

The primary sources of data for this study were government documents such as the Statistical Yearbook of the Ministry of Health (MOH) and the General Authority of Statistics (GAS) [11,15,16,17,18,19]. These documents provide statistics on the number of malaria cases recorded in 20 region (see Table 1 and Table 2), as well as population estimates for each region. Malaria cases in these documents are reported for *P. falciparum* malaria cases as well as the sum of *P. vivax* and *P. ovale* malaria cases. We explored the *P. vivax* and *P. ovale* malaria distributions as one in this work. Moreover, ecological data such as temperature and rainfall were collected from publicly available sources to investigate the influence of environmental variables on the distribution of malaria in the KSA. Each region’s yearly population estimates were extracted from the 2017–2021 population census [15].

### 2.3. Statistical Methods

The statistical analysis comprised four steps. First, the crude yearly incidence rate of malaria in each region was calculated by dividing the total number of new malaria cases by the population of the same year in the corresponding region, multiplied by 100,000 to obtain a rate per 100,000 population. Second, the standardized incidence ratio (SIR) was calculated to estimate the relative risk of malaria in each region. For each region i, i = 1…n, the SIR was calculated as the ratio of the observed number of malaria cases in the region (Y_*i*_) to the expected number of malaria cases (E_*i*_) in the region across the study period: SIR_*i*_ = Y_*i*_/E_*i*_. The expected count E_*i*_ represented the total number of malaria cases that one would expect if the population of region i had the same risk as the national population. The expected number of malaria cases for each region (E_*i*_) was computed as: E_*i*_ = $r_{j}^{\left(s\right)}$ $n_{j}^{\left(s\right)}$, where $r_{j}^{\left(s\right)}$ is the overall crude malaria incidence rate for the KSA (i.e., total number of malaria cases divided by total population in all regions), and $n_{j}^{\left(s\right)}$ is the population of each region i. Third, we conducted a geospatial analysis using the georeferenced data that were linked to the region SIR*_i_*. Choropleth maps of SIRs were developed to assess the spatial distributions of malaria incidence at the region level. The SIR was measured for *P. falciparum* malaria cases as well as the *P. vivax* and *P. ovale* malaria cases.

The standardized incidence ratio is a typical metric for comparing the disease incidence of a cohort to that of the general population. A SIR higher (or less than) 1 implies that there are more (or fewer) observed cases than expected based on population size and structure. The numerical value of the SIR can be used to assess the size of the difference in different districts. SIR values were broken down into six categories in this study for clarity and comprehension. A region is considered high-risk, higher-risk, or highest-risk if its SIR values are within the range 1 < SIR < 2, 2 < SIR < 4, or 4 < SIR, respectively. A region is low-risk, lower-risk, or lowest-risk if its SIR values are within the range 0.5 < SIR < 1, 0.25 < SIR < 0.5, or SIR < 0.25, respectively. Fourth, to examine the heterogeneity of malaria endemicity in the KSA, we estimated the proportion of *P. falciparum* malaria cases and *P. vivax* and *P. ovale*, respectively, to the total number of malaria cases per region in the KSA from 2017 to 2021. The percentage ratio (R) was calculated using the formula R_*i*_ = Y_*i*_/T_*i*_%, where Y_*i*_ represents the number of cases of either *P. falciparum* malaria or *P. vivax* and *P. ovale* in region i, and T_*i*_ represents the total number of malaria cases in that region.

## 3. Results

Table 1 provides some statistics on the malaria cases in the KSA, including total malaria cases, cases per species, and cases stratified by age group in the study period. During the five-year span, the total cases varied between two to three thousand, with the minimum in 2019 with a total of 2152 cases and the maximum in 2020 with a total of 3658 cases. This increase can be attributed to COVID-19-related service interruption in the KSA. The majority of positive malaria cases were due to *P. falciparum*, ranging from 66 to 88% during the study period, and the rest of the malaria cases were due to *P. vivax* and *P. ovale*. Furthermore, during the study period, the majority of malaria infections (95%) were among the age group of 10 years and above. Table 2 provides the incidence rate per 100,000 for each species in the KSA in the period between 2017 and 2021. The two regions with the consistently high incidence rate were Jazan for *P. falciparum* malaria cases and Najran for *P. vivax* and *P. ovale* infections.

**Figure 1:**
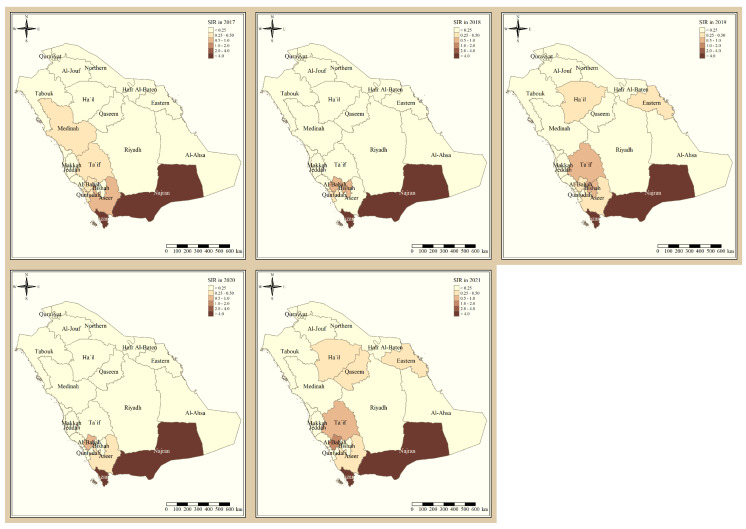
The standardized incidence ratio of *P. falciparum* malaria cases in the KSA between 2017 and 2021.

**Figure 2:**
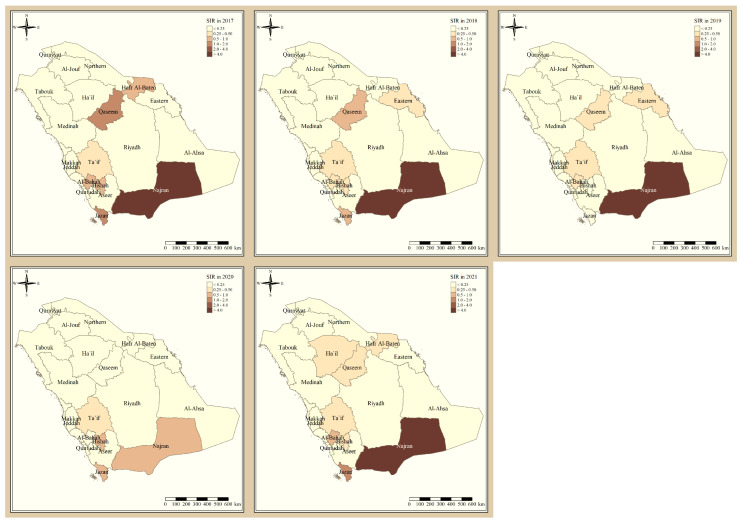
The standardized incidence ratio of *P. vivax* and *P. ovale* malaria cases in the KSA between 2017 and 2021.

Figure 1 shows the standardized incidence ratio of *P. falciparum* malaria cases in the KSA between 2017 and 2021. Since 2017, the total number of high-risk regions remained constant, 4 regions, and were all located in the southern part of the country. The highest-risk regions for *P. falciparum* malaria were Jazan and Najran, which were consistently endemic throughout the five years of this study. Al-Bahah, Taif, Aseer, and Bishah are regions in the south and south-west of the Kingdom that had varied levels of *P. falciparum* endemicity fluctuating between high- and low-risk regions. The remaining northern and eastern parts of the country are considered very low risk and have been since 2017. Figure 2 shows the standardized incidence ratio of *P. vivax* and *P. ovale* malaria cases in the KSA between 2017 and 2021. The distribution of high-risk regions is more heterogeneous compared to that of *P. falciparum* in the KSA, whereby high-risk regions extend beyond the southern states. The number of high-risk regions dropped by half from six in 2017 (Najran, Jazan, Al-Bahah, Bishah, Qaseem, and Hafr Albaten) to only three regions in 2021 (Jazan, Najran, and Al-Bahah). The highest-risk regions for *P. vivax* and *P. ovale* malaria are similarly Jazan and Najran, which are consistently endemic throughout the five years of this study. Al-Bahah, Bishah, Qaseem, and Hafr Albaten are regions with varied levels of *P. vivax* and *P. ovale* endemicity, fluctuating between high and low risk. The remaining seven regions in the Kingdom are considered very low risk for *P. vivax* and *P. ovale* infection and have been since 2017.

**Figure 3:**
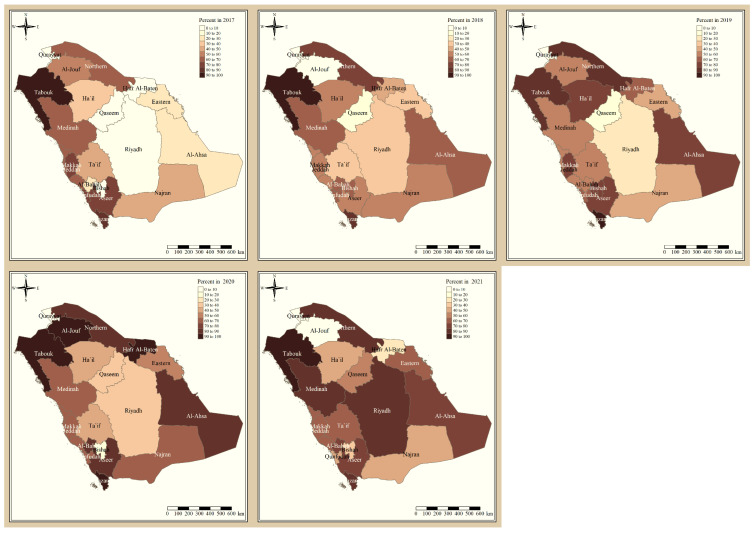
The percentage of *P. falciparum* malaria cases to the total number of cases in the KSA between 2017 and 2021.

**Figure 4:**
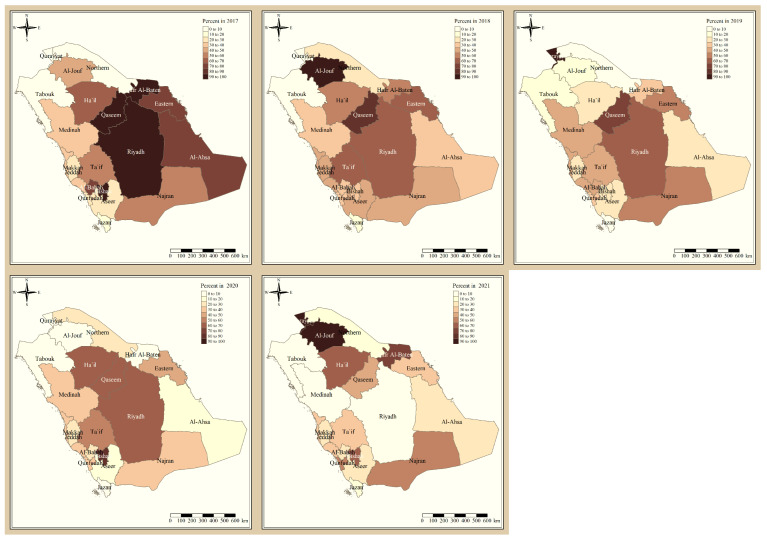
The percentage of *P. vivax* and *P. ovale* malaria cases to the total number of cases in the KSA between 2017 and 2021.

**Figure 5:**
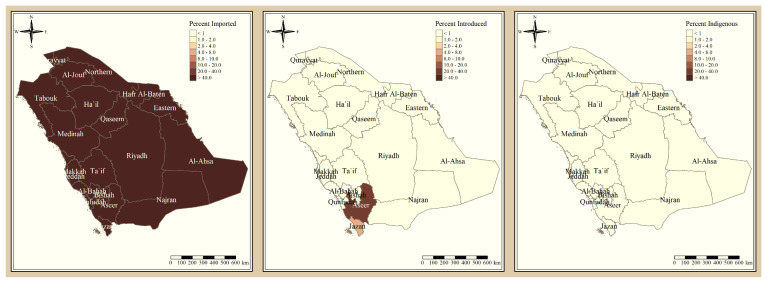
The percentage of malaria source to the total number of cases in the KSA in 2021.

**Figure 6:**
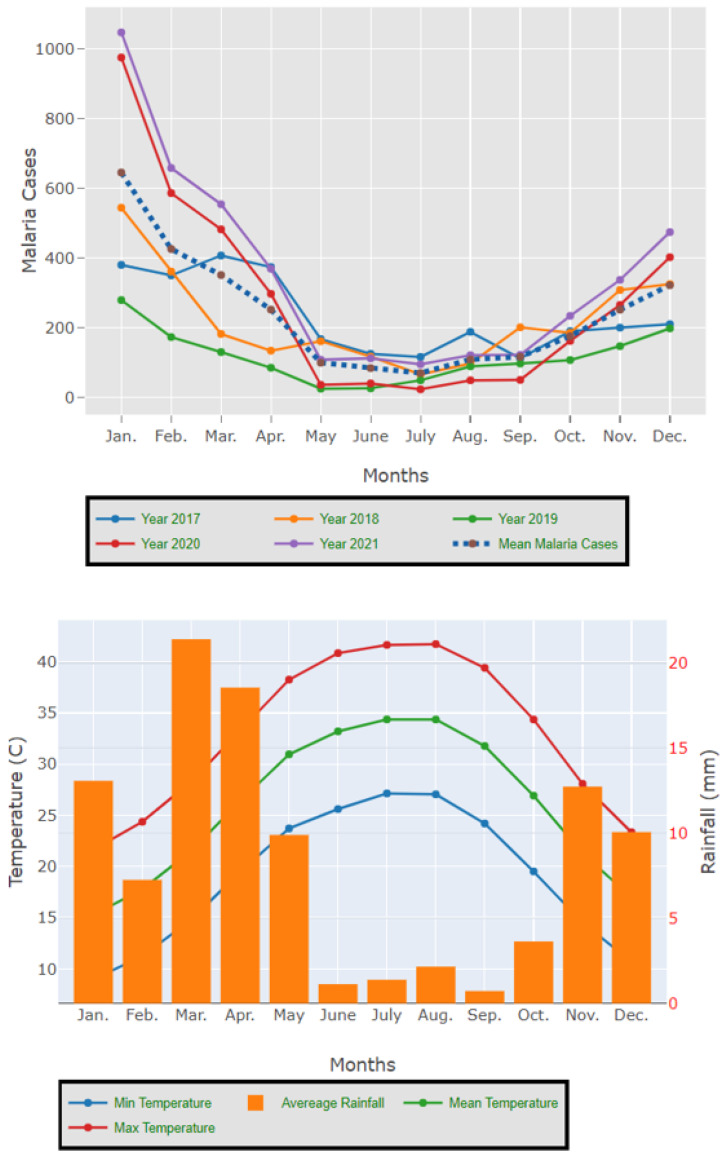
The seasonal variation in malaria cases in the KSA between 2017 and 2021 as well as the temperature and rainfall average for the five years.

The results presented in Figure 3 and Figure 4 illustrate the proportion of *P. falciparum* malaria cases and *P. vivax* and *P. ovale*, respectively, out of the total number of malaria cases per region in the KSA from 2017 to 2021. The data indicate that Jazan has the highest percentage of *P. falciparum* infections compared to other malaria species, indicating that *P. falciparum* is the dominant parasite in this region. In contrast, the central regions (Riyadh, Qassem, and Ta’if) in the KSA display a fluctuating percentage of species infections, ranging from high *P. falciparum* and low *P. vivax* and *P. ovale* in one year to the opposite in a different year, with *P. vivax* and *P. ovale* dominating overall in the five years of this study. The results presented in Figure 5 illustrate the proportion of malaria cases in the KSA in 2021 that were either locally introduced or imported. Notably, the data show that no local (indigenous) cases were reported during this period, with only Jazan and Aseer regions reporting introduced cases. The remaining regions had imported cases, indicating the potential for malaria transmission from external sources. The results presented in Figure 6 illustrate the seasonal patterns of malaria cases in the KSA from 2017 to 2021 (lower panel) as well as the average temperature and rainfall over the same period (upper panel). The figure demonstrates that the number of malaria cases starts to increase in November and reaches its peak in January, which coincides with a period of increased rainfall and decreased temperature.

## 4. Discussion

Since 2017, the total number of high-risk regions (four) remained constant, and all were located in the southern part of the country. The highest-risk regions for *P. falciparum* malaria are Jazan and Najran, which were consistently endemic throughout the five years of this study. This result is similar to the study that found that Jazan region had most of the *P. falciparum* infection cases [9]. This outbreak might have been impacted by increased cross-border malaria importation [20], limited public awareness of malaria management techniques [21], parasite resistance to medication, and vector resistance to insecticides.

Al-Bahah, Aseer, and Bishah are regions in the south and south-west of the Kingdom with varied levels of *P. falciparum* endemicity fluctuating between high- and low-risk regions. The remaining Northern and Eastern parts of the country are considered very low risk and have been since 2017, and these results are supported by a report from MOH [11]. The distribution of high-risk regions is more heterogeneous compared to that of *P. falciparum* in the KSA, whereby high-risk regions extend beyond the southern states. The number of high-risk regions dropped by half from six in 2017 (Najran, Jazan, Al-Bahah, Bishah, Qaseem, Hafr Albaten) to only three regions in 2021 (Jazan, Najran and Al-Bahah). This could be due to the concerted elimination strategy in the country [11]. The highest risk regions for *P. vivax* and *P. ovale* malaria are similarly Jazan and Najran, which were consistently endemic throughout the five years of this study. Al-Bahah, Bishah, Qaseem, and Hafr Albaten are regions with varied levels of *P. vivax* and *P. ovale* endemicity fluctuating between high and low risk, similar to the study in 2021 [8,11]. The remaining seven regions in the Kingdom are considered very low risk for *P. vivax* and *P. ovale* infection and have been since 2017.

The study indicates that *P. falciparum* is the dominant malaria parasite in Jazan, aligning with a previous study reporting a 94.3% infection rate [9]. This underscores the necessity for focused treatments to control and prevent *P. falciparum* in Jazan, which, while not yet eliminated, has achieved notable public health advancements [22]. In contrast, the central regions of the KSA (Riyadh, Qassim, and Ta’if) exhibited varying percentages of malaria species infections, with *P. vivax* and *P. ovale* dominating over the five-year study. These findings highlight the inconsistency in malaria species incidence across KSA regions, emphasizing the need for tailored interventions based on species prevalence. The 2021 findings for malaria cases in the KSA revealed that all reported cases were either locally introduced or imported. Notably, no indigenous cases were reported, with only Jazan and Aseer having introduced cases, while all other regions had imported cases [19,22]. This highlights the persistent challenge of imported malaria in the country, particularly among migrant workers from East Africa, Yemen, and South Asia. These results align with previous research indicating a consistent flow of imported malaria cases in the KSA [9,23,24]. Our results suggest a strong association between malaria transmission and climatic conditions, particularly temperature and rainfall [9,25]. These factors are known to influence the life cycle of mosquitoes and the development of the malaria parasite. The peak in malaria cases during November and January could be attributed to the breeding of mosquitoes in stagnant water pools caused by heavy rainfall.

Individual migration has been found to rapidly undermine malaria control successes, and imported malaria remains a severe problem in the KSA. The border with Yemen is especially concerning, and malaria treatment in this region presents Saudi Arabia with a significant challenge in achieving and maintaining malaria-free status. The number of imported cases is increasing in the southern districts. This can be attributed in part to a halt in malaria control activities on the Yemeni side of the border, as well as the Yemeni war, which has resulted in increasing legal and illegal immigration into the KSA [20]. Malaria importation will continue, and the presence of vectors will result in local transmission. To mitigate this risk, the KSA must develop ways to limit endemic transmission.

Malaria infections caused by *P. vivax* increased in 2021; hence, Saudi Arabia may face new challenges. *P. vivax* is more difficult to control than *P. falciparum* for a number of reasons, including the fact that its dormant liver stages can lead to relapses, its extrinsic incubation period is shorter, parasite densities are frequently lower than the level detectable by diagnostic tests, and compliance with the 7–14 day primaquine regimen for treating liver stages is frequently poor [26]. These factors make detecting and managing *P. vivax* residual transmission more challenging. To address the rising incidence of *P. vivax* infections, new and simple methods for identifying and treating people infected with the parasite are needed. This might entail using sensitive, portable diagnostic tools like a loop-attenuated isothermal amplification test [27]. This test is as sensitive as PCR, but it may be used in remote areas and in the field [27].

The KSA has taken impressive steps towards the goal of malaria eradication, but it is crucial to sustain these efforts to ultimately achieve elimination. The process of eradicating malaria in the KSA must be comprehensive, encompassing all aspects of the problem. This includes the establishment of reliable surveillance and early detection systems to detect cases of malaria promptly and to respond quickly to outbreaks. Vector control measures such as the use of insecticide-treated bed nets and indoor residual spraying should also be implemented to minimize the risk of transmission. Provision of treatment and prophylaxis to infected individuals is crucial, as is promoting health education to increase awareness among the public. To address cross-border transmission, the KSA should collaborate with neighboring countries. The government and healthcare organizations have a critical role to play in effectively managing and coordinating the malaria eradication program, which requires investment in research and development of new diagnostic tools and treatments. The use of modern disease surveillance systems will help in guiding the elimination interventions and ensuring their effectiveness. It is imperative to keep in mind that malaria eradication is not a one-time event but a continuous process that requires sustained effort and commitment from all relevant stakeholders. This requires ongoing efforts from various actors, including the government, healthcare organizations, and the public, to ensure the eradication of malaria in the KSA. The investment in research and development of new tools and treatments is crucial in achieving this goal, as it helps to stay ahead of the disease and continue making progress towards elimination.

While the current study provides valuable insights into the epidemiology, distribution, trends, and ecological determinants of malaria risk in the KSA between 2017 and 2021, it has some limitations that should be considered when interpreting the findings. One of the main limitations of this study is its reliance on secondary data from the Ministry of Health, while these data are useful for identifying trends and patterns, it may have limitations in terms of completeness. Moreover, the study did not consider potential confounding factors such as socioeconomic status and access to healthcare, which could have influenced malaria risk in different regions of the country. Despite these limitations, the study’s comprehensive approach and large dataset covering multiple regions and a long period of time are significant strengths. This allows for a broader understanding of malaria risk factors and trends across the country. Additionally, the use of secondary data provided a cost-effective and efficient means of analysis. Therefore, while caution should be taken when interpreting the results of this study due to its limitations, the findings contribute to the current knowledge on malaria in the KSA and provide a foundation for future research and public health interventions.

## 5. Conclusions

This study provides valuable insights into the epidemiology, distribution, trends, and ecological determinants of malaria risk in the KSA between 2017 and 2021. The study sheds light on the current state of malaria in the Kingdom, highlighting the number of reported cases, the species of *Plasmodium* responsible for the majority of cases, and the regions where the disease remains endemic. Environmental factors, such as temperature and rainfall, have been shown to play a crucial role in the distribution of malaria in the KSA. The findings of this study will be of significant importance in ongoing efforts to control and eliminate malaria in the KSA. The study has highlighted the need for targeted interventions, including vector control measures, prompt diagnosis and treatment of cases, and improved access to healthcare in malaria-endemic areas. Furthermore, data from this study highlight the importance of ongoing surveillance and monitoring of malaria trends, as well as the need for further research to better understand the complex ecological factors that contribute to the transmission and distribution of malaria in the KSA.

## Figures and Tables

**Table 1:** The total number of malaria cases, cases due to *P. falciparum* malaria (Pf), *P. vivax* (Pv), and *P. ovale* (Po) as well as the age-stratified malaria cases in the KSA in the period between 2017 and 2021.

Year	Total Malaria Cases	Total Pf Cases	Total Pv + Po Cases	Percent of Cases in Ages 0–10 Years Old	Percent of Cases in Ages >10 Years Old
2017	2715	1816	885	4.23%	95.77%
2018	2711	1898	802	1.43%	98.57%
2019	2152	1498	628	2.14%	97.86%
2020	3658	3231	419	0.62%	99.38%
2021	2616	2023	480	2.34%	97.66%

**Table 2:** The incidence rate per 100,000 of *P. falciparum* malaria (Pf), *P. vivax* (Pv), and *P. ovale* (Po) in the KSA in the period between 2017 and 2021.

Region	2017		2018		2019		2020		2021	
	**Pf**	**Pv + Po**	**Pf**	**Pv + Po**	**Pf**	**Pv + Po**	**Pf**	**Pv + Po**	**Pf**	**Pv + Po**
Riyadh	0.11	1.15	0.42	0.77	0.32	0.79	0.14	0.24	0.83	0.10
Makkah Al Mukarramah	1.59	0.47	0.36	0.30	0.62	0.23	0.24	0.10	0.21	0.09
Jeddah	3.07	1.92	2.34	2.07	2.81	2.62	1.94	1.13	2.02	1.11
Ta’if	5.66	6.24	3.05	4.94	8.28	6.24	2.61	3.19	6.39	3.34
Al Madinah Al Munawwarah	4.20	2.26	2.91	1.29	2.45	1.84	0.58	0.29	1.55	0.16
Eastern	0.20	0.61	2.95	6.08	3.57	4.80	1.70	1.43	4.15	1.87
Al-Ahsa	1.03	3.10	1.22	0.75	2.92	0.94	1.60	0.28	2.16	0.66
Hafr Al-Baten	0	6.70	1.83	2.03	1.83	1.02	1.83	0	1.42	3.45
Qaseem	1.58	16.08	1.44	7.04	1.15	5.31	1.01	1.87	4.02	3.30
Aseer	7.88	2.13	1.76	1.50	3.63	1.04	4.88	0.99	3.42	1.04
Bishah	0.49	6.33	6.33	3.41	6.33	1.95	1.46	8.28	2.43	4.87
Tabuk	0.23	0	0.34	0	2.28	0.46	0.23	0	0.80	0
Ha’il	0.40	0.81	2.02	2.02	4.04	1.62	0.81	1.21	3.23	4.85
Northern	2.97	0	1.98	0.66	2.97	0	1.32	0.33	1.65	0.33
Jazan	75.09	12.73	93.46	10.87	55.75	0.55	195.53	12.46	94.63	13.08
Najran	64.10	90.33	43.71	40.79	40.79	43.71	26.22	11.66	46.62	61.19
Al Bahah	3.84	10.55	7.67	3.84	4.80	3.84	11.51	2.88	23.98	6.71
Al Jouf	0.98	0.79	0	0.79	0.59	0.20	0.20	0	0	0.39
Qurayyat	0	0	0	0	0	1.61	0	0	0	0.54
Qunfudah	4.66	1.33	4.33	2.00	2.33	1.33	1.33	0.67	1.00	1.33

## Data Availability

All the data used in this research were obtained from the KSA’s Ministry of Health https://www.moh.gov.sa/en/Pages/default.aspx (accessed on 1 March 2023) and the General Authority of Statistics https://www.stats.gov.sa/en (accessed on 1 March 2023).
